# Thermo-optic phase shifters based on silicon-on-insulator platform: state-of-the-art and a review

**DOI:** 10.1007/s12200-022-00012-9

**Published:** 2022-04-12

**Authors:** Shengping Liu, Junbo Feng, Ye Tian, Heng Zhao, Li Jin, Boling Ouyang, Jiguang Zhu, Jin Guo

**Affiliations:** Chongqing United Microelectronics Center, Chongqing, 401332 China

**Keywords:** Thermo-optic phase shifter, Photonic integrated circuits (PICs), Optical switches, Silicon photonics

## Abstract

Silicon photonic platforms offer relevance to large markets in many applications, such as optical phased arrays, photonic neural networks, programmable photonic integrated circuits, and quantum computation devices. As one of the basic tuning devices, the thermo-optic phase shifter (TOPS) plays an important role in all these applications. A TOPS with the merits of easy fabrication, low power consumption, small thermal time constant, low insertion loss, small footprint, and low crosstalk, is needed to improve the performance and lower the cost of the above applications. To meet these demands, various TOPS have been proposed and experimentally demonstrated on different foundry platforms In this paper, we review the state-of-the-art of TOPS, including metal heater, doped silicon, silicide, with silicon substrate undercut for heat insulation, folded waveguide structure, and multi-pass waveguide structure. We further compare these TOPSs and propose the directions of the future developments on TOPS.

**Graphical abstract:**

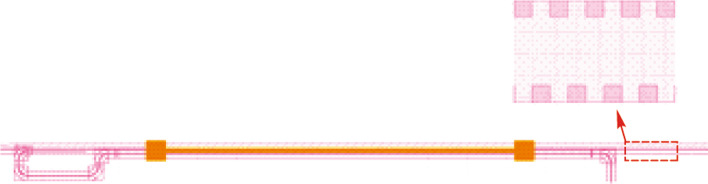

## Introduction

Benefiting from the complementary metal–oxide–semiconductor (CMOS) compatibility, silicon photonics is becoming a key technology for implementing high-density photonic integrated circuits (PICs) with complex functionalities [1–3]. The functions of these PICs are usually achieved through phase shifters [4, 5]. Tuning principles of phase shifters are mainly based on the thermo-optic effect, the electro-optic effect, or the nano-opto-electro-mechanical effect [6–8]. Some other types of phase shifters have also been proposed and used extensively [9, 10]. The thermo-optic coefficient of silicon in the C band over the temperature range 300–600 K can be written as [11]

$$\frac{{{\text{d}}n}}{{{\text{d}}T}} = 9.45 \times 10^{ - 5} + 3.47 \times 10^{ - 7} \times T - 1.49 \times 10^{ - 10} \times T^{2} \left( {{\text{K}}^{ - 1} } \right).$$

Therefore, the refractive index of a waveguide can be changed through a thermo-optic phase shifter (TOPS). The values of thermo-optic coefficient and heat conductivity are about 1.8 × 10^−4^ K^–1^ and 149 W/mK, respectively [12]. Compared with the TOPS, electro-optic phase shifters always have a greater modulation bandwidth due to the free-carrier plasma dispersion effect. However, these devices usually suffer from substantial insertion loss due to free-carrier absorption [13]. Although the phase shifter based on the nano-opto-electro-mechanical effect has low power consumption, it is hard to fabricate. In addition, this type of phase shifter is liable to break down due to mechanical fatigue [14].

As a result of simple design, easy fabrication, low cost, and small footprint, the TOPS is widely used for photonic devices and large-scale integrated PICs on the silicon-on-insulator (SOI) platform [15–17]. Typical photonic devices that use the TOPS are Mach–Zehnder interferometer (MZI) [18–20], micro-disk [21], and micro-ring resonator (MRR) [22–24]. The TOPS in these photonic devices is used to change the phase of light by locally controlling the temperature in the phase-shifting region with TOPS. However, it is worth mentioning that the modulation bandwidth of TOPS is less than one hundred of kilohertz, the TOPS is only suitable for applications that do not require high modulation speed [25–28]. Regarding these characteristics of TOPS, monolithic integrated PIC with TOPS have been applied in some special applications, such as optical neural networks [29–31], quantum photonic devices [32–34], optical phased array [35, 36], reconfigurable optical add-drop multiplexers (ROADMs) [37], programmable photonic circuit [38–40], and thermally-tunable optical delay lines [41]. The requirements of these PICs for TOPS will be discussed in Sect. 4.

Many researchers are working toward improving the performance of TOPS [42–45]. Methods including air-gap trench or silicon substrate undercut post-processing, folded waveguide, and multi-pass waveguide have been proposed and demonstrated. A trade-off between the power consumption and the thermal time constant has also been investigated [46, 47]. In this paper, we give an overview of the current status of the TOPS based on silicon photonics technologies. More specifically, we focus on the TOPS that is ready for massive application and fabricated in foundry platforms, including IMEC, AMF, IBM, OpSIS, CUMEC, and so on, by a standard silicon fabrication process, We also discuss the outlook for further development of TOPS, at the end of this paper.

## Principle of TOPS

**Fig. 1:**
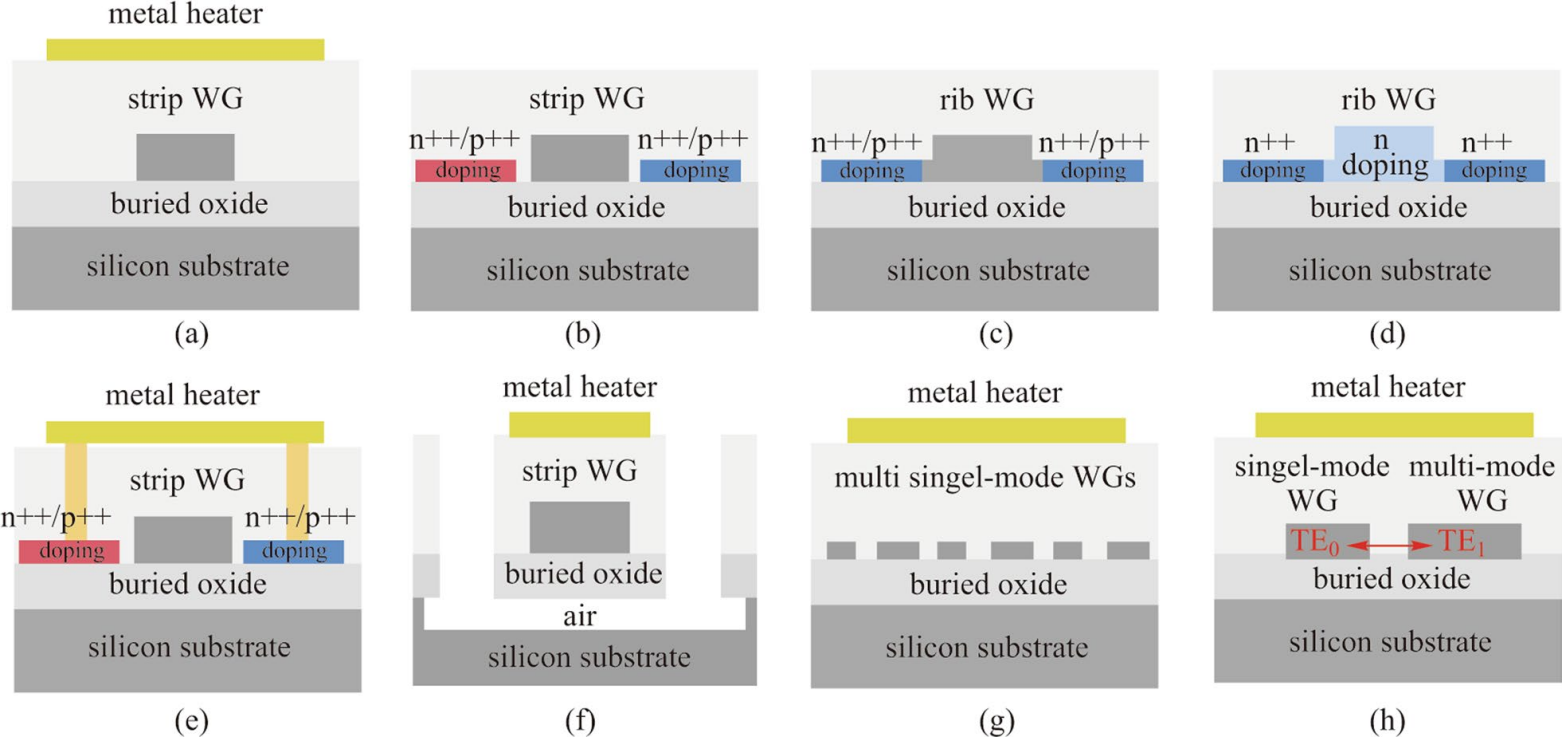
Cross-sections of different kinds of TOPS. **a** TOPS based on strip waveguide with a metal heater on the top. **b** TOPS based on strip waveguide with doped-silicon heaters at both sides. **c** TOPS based on rib waveguide with doped-silicon heaters at both sides. **d** TOPS based on rib waveguide with a directly integrated doped silicon heater. **e** TOPS based on strip waveguide surrounded by a hybrid heater. **f** TOPS with air-gap trench and silicon substrate undercut. **g** TOPS based on the folded waveguide with a metal heater on the top. **h** TOPS based on multi-pass waveguides with a metal heater on the top

A TOPS is composed of a waveguide structure and a resistive heater. As shown in Fig. 1a and c, the shape of a waveguide on the SOI platform can be a strip or rib structure. Typically, both of them consist of a 2.0 μm silica lower cladding, 220 nm silicon core, and 2.0 μm silica upper cladding. Figure 1a–h provides the cross-sections of different kinds of TOPS, which will be discussed in detail in Sect. 3.

Due to the wide variety of materials and design complexities offered by CMOS technologies, the material used for the resistive heater can be doped silicon, silicide, or metal wiring. The line resistivity and fabrication process of the three types of heaters are different, which makes it possible to design heaters with different dimensions.

Generally, the amount of phase shift caused by resistive heater can be expressed as [48]

1 $$\Delta \varphi = \frac{{2{\uppi }}}{\lambda }\left( {\frac{{{\text{d}}n_{{{\text{eff}}}} }}{{{\text{d}}T}}} \right)\Delta TL,$$

where *λ* is the wavelength of the light, ${{{\text{d}}n_{{{\text{eff}}}} } \mathord{\left/ {\vphantom {{{\text{d}}n_{{{\text{eff}}}} } {{\text{d}}T}}} \right. \kern-\nulldelimiterspace} {{\text{d}}T}}$ is the thermo-optic coefficient of the silicon waveguide, *L* is the length of TOPS, and Δ*T* denotes the change of temperature. Since Δ*T* is caused by the action of the resistive heater, it can be written as

2 $$\Delta T = \frac{\eta P}{{C_{p} \rho LS}}.$$

Here, *η* is the utilization tuning efficiency of drive power, *P* is the power consumed by the resistive heater, *C*_*p*_ is the heat capacity of the waveguide, *ρ* is the material density of the waveguide, and *S* is the cross-sectional area of the waveguide. The amount of phase shift can be written as

3 $$\Delta \varphi = \frac{{2{\uppi }}}{\lambda }\left( {\frac{{{\text{d}}n_{{{\text{eff}}}} }}{{{\text{d}}T}}} \right)\frac{\eta P}{{C_{p} \rho S}}.$$

The tuning efficiency of TOPS is usually expressed in terms of electrical power needed for a π phase shift (*P*_π_), which can be expressed as

4 $$P_{{\uppi }} = \frac{\lambda }{2}\left( {\frac{{{\text{d}}T}}{{{\text{d}}n_{{{\text{eff}}}} }}} \right)\frac{{C_{p} \rho S}}{\eta }.$$

Therefore, the tuning efficiency of TOPS is mainly determined by the utilization tuning efficiency of drive power. An effective way to improve the tuning efficiency of TOPS is reducing heat leakage to the environment.

In addition to tuning efficiency, the thermal time constant is also an important factor for TOPS. The thermal thermal time constant of TOPS can be written as

5 $$\tau = {H \mathord{\left/ {\vphantom {H G}} \right. \kern-\nulldelimiterspace} G},$$

where *H* is the heat capacity of the heated waveguide, and *G* denotes the thermal conductance of the waveguide to the environment. *H* and *G* can be expressed as

6 $$\left\{ \begin{gathered} H = C_{p} \rho LA, \hfill \\ G = P_{{\uppi }} /\Delta T_{{\uppi }} . \hfill \\ \end{gathered} \right.$$

Here, *A* is the area of heat flow. By substituting Eqs. (2) and (6) into Eq. (5), the thermal time constant can be rewritten as

7 $$\tau = \frac{\eta A}{S}.$$

Therefore, the thermal time constant is influenced by the utilization efficiency, the area perpendicular to the direction of heat flow, and the cross-sectional area of the waveguide. Moreover, the product of thermal time constant and power consumption, i.e., the figure of merit (FOM), can be expressed as

8 $$P_{{\uppi }} \tau = \frac{{\lambda C_{p} \rho A}}{2}\frac{{{\text{d}}T}}{{{\text{d}}n_{{{\text{eff}}}} }},$$

which can be reduced by decreasing the area perpendicular to the direction of heat flow, such as by directly integrating a doped silicon heater with a waveguide. However, the insertion loss of this kind of TOPS is relatively large, which is not suitable for large-scale networks. In addition, the TOPSs with folded and multi-pass waveguide have been proposed to reduce power consumption. Adversely, this type of structure would increase the size of the footprint and insertion loss. The pros and cons of all these TOPS devices will be described in the next section.

## Comparison of different TOPS

Many studies have focused on improving the characteristics of TOPS, such as by increased tuning efficiency, faster thermal time constant, lower insertion loss, and smaller footprint. According to the difference between the structural differences of these TOPSs they can be classified as a basic structure, silicon substrate undercut, folded waveguide, multi-pass waveguide, and integrated with diode.

### TOPS with a basic structure

**Fig. 2:**
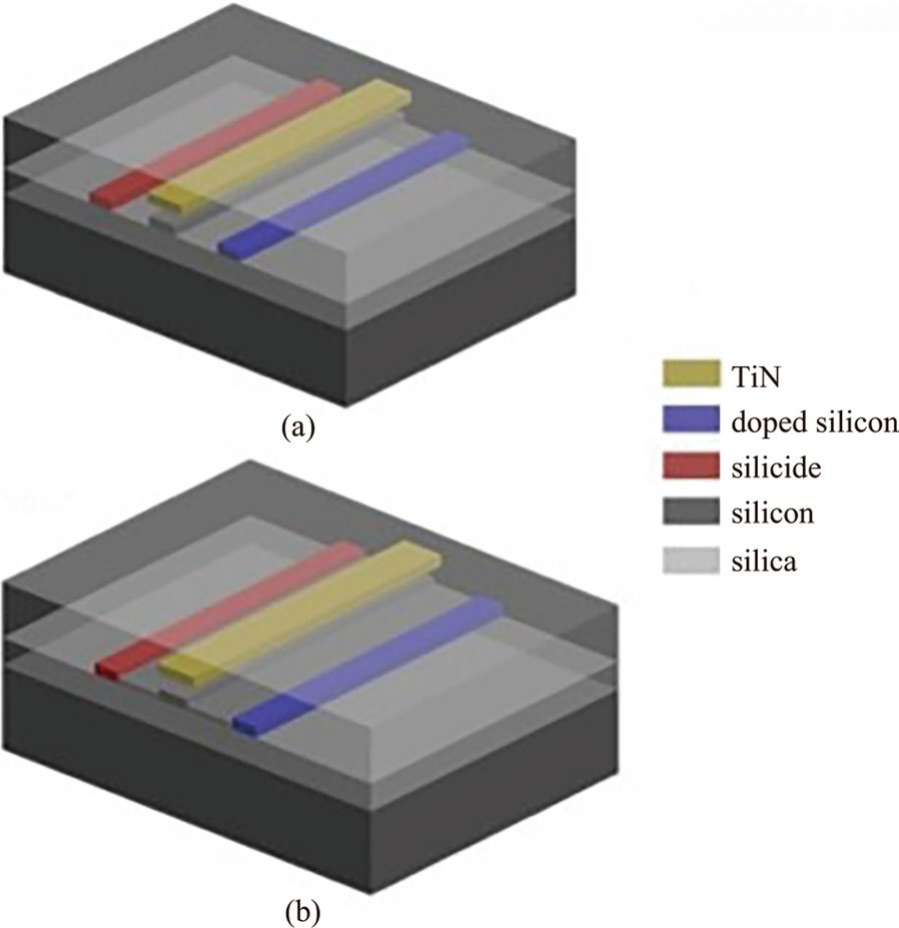
Different TOPS of **a** strip waveguide and **b** rib waveguide with a basic structure based on SOI platform

For a basic TOPS structure, a resistive heater of doped silicon or silicide is placed on both sides of a waveguide. At the same time, a metal resistive heater can be fabricated above the waveguide, as shown in Fig. 2. It needs to be mentioned that the resistivity of the heater line should be much larger than that of connecting wire, which is usually made of metal aluminum (Al) or copper (Cu).

Almost all silicon foundry platforms can manufacture these types of TOPSs, whose performances are shown in Table 1. The columns include heater type, waveguide type, test structure, tuning efficiency, heating time, cooling time, and foundry.

**Table 1:** Performance list of conventional TOPS fabricated on different foundries

Heater type	Waveguide type	Test structure	Tuning efficiency/(mW·π^–^^1^)	Heating time/μs	Cooling time/μs	Foundry	References
TiN	Single strip WG	MZI	19.12	5.90	8.97	CUMEC	[49]
Silicide	Single strip WG	MZI	21.75	12.90	4.0	CUMEC	[49]
Doped silicon	Single strip WG	MZI	21.96	12.80	2.60	CUMEC	[49]
Tungsten	Single strip WG	MZI	23.4	38.2	45.11	IMEC	[50]
Silicide	Single strip WG	MZI	22.5	19.1	75.8	IMEC	[50]
Doped silicon	Single strip WG	MZI	20.4	21.3	66.0	IMEC	[50]
TiN	Single strip WG	MZI	21.4	5.6	NR	AMF	[15]
Silicide	Single rib WG	MZI	20	2.8	2.2	IBM	[16]
Doped silicon	Single rib WG	MZI	24.77 ± 0.43	NR	NR	OpSIS	[51]

TiN: titanium nitride, WG: waveguide, NR: not reported, MZI: Mach–Zehnder interferometer

The tuning efficiency of all these TOPS with a basic structure is about 20 mW/π. The minor differences among tuning efficiency and thermal time constant of these TOPS might be caused by the material characteristics, processing technology, and testing equipment. The characteristics of these TOPS fabricated on CUMEC and other foundries are on the same level.

Moreover, the drive voltage of TOPS can be written as [16]

9 $$V = \sqrt {PR} .$$

**Fig. 3:**
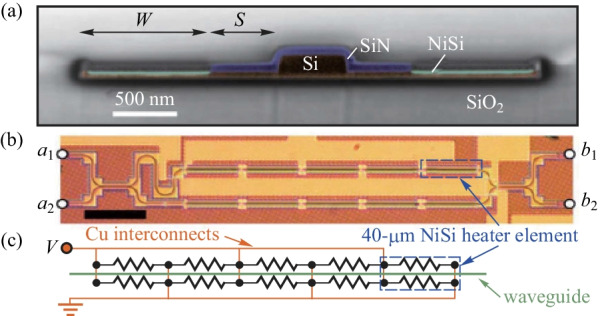
TOPS with an electrically parallel heater [16]. **a** Scanning electron micrograph (SEM) cross-sectional image of an SOI rib waveguide with a heater of silicide. **b** Heater of silicide was implemented into the MZI. **c** Electrical configuration of the heater of silicide in the upper arm of the MZI

Here, *P* is the drive power of TOPS, and *R* denotes the resistance of the heater in TOPS. To use CMOS compatible drive voltage, which is always less than 1.0 V, a heater of silicide elements is electrically connected in parallel utilizing Cu connections (see Fig. 3). The resistance can be small enough, using the parallel connection, to apply a low drive voltage [16]. As a result, this kind of TOPS operates with the tuning efficiency of *P*_π_ = 20 mW/π and a thermal time constant of *τ* < 2.8 μs, using a 1 V drive voltage. Unfortunately, the excess optical loss is about 25 dB/cm, which is caused by scattering and absorption loss [16].

**Fig. 4:**
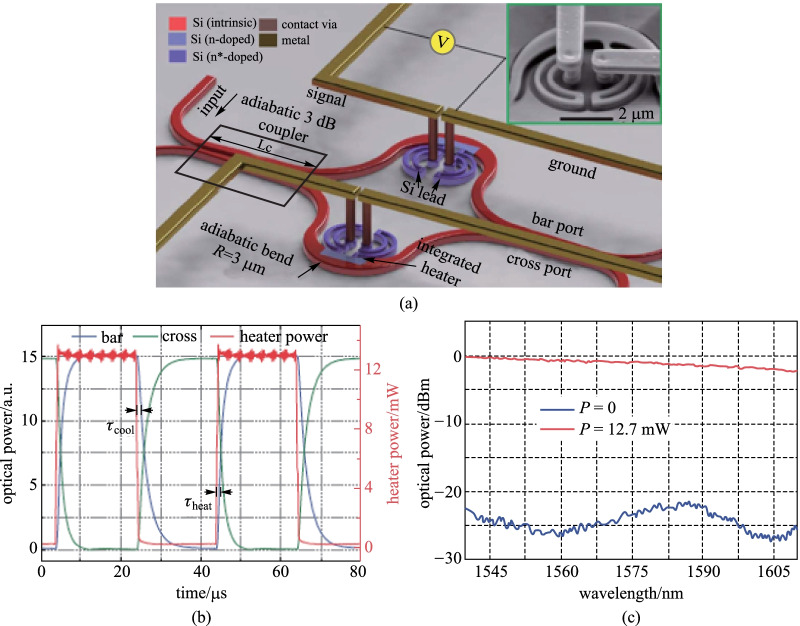
TOPS with a directly integrated silicon heater [48]. **a** Schematics of the proposed structure. Inset: SEM image of the fabricated optical switch. **b** Time-domain measurement of the fabricated TOPS under an electrical consumption power of 12.7 mW, with a heating time and cooling time of 2.2 and 2.4 μs, separately. **c** Frequency domain measurement in the cross port showing a bandwidth of 70 nm and extinction of  > 20 dB

In addition, a TOPS based on a strip waveguide with a directly integrated doped silicon heater has been demonstrated by Watts et al. [48], as shown in Fig. 4. Although the thermal time constant is the same as the TOPS with an electrically parallel heater, the tuning efficiency is only 12.7 mW/π due to the silicon waveguide core itself being used as a resistive heater. The insertion loss of the TOPS with a directly integrated doped silicon heater is about 0.5 dB, which is not beneficial for a large-scale PIC.

**Fig. 5:**
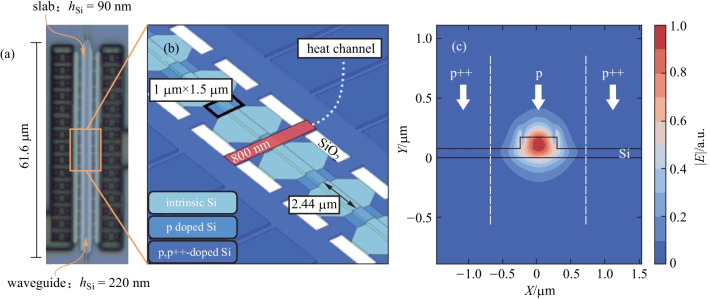
A low-loss TOPS with a directly integrated silicon heater [51]. **a** Optical micrograph of the structure. **b** Perspective view of the structure. **c** Doping profile along the cross-section marked red in (**b**), overlapped with the simulated amplitude of the horizontal component of the electrical field

Due to the fact that insertion loss of a TOPS with a directly doped silicon heater in Ref. [48] is high, a novel TOPS with low-loss has been proposed and experimentally proved by Harris et al. [51]. As shown in Fig. 5b, there is only an 800 nm wide channel connecting the contact region to the ridge waveguide, which efficiently restricts the outward propagation of heat. There is sufficient clearance between the guiding region and the p++-doped region of 2.44 μm to avoide insertion loss due to the free-carrier absorption. A 61.6 μm long TOPS is fabricated with a propagation loss of (0.23 ± 0.13) dB for 21 devices. At the same time, the *P*_π_ of the device is (24.77 ± 0.43) mW/π and the thermal time constant of the device is 2.69 μs, which is similar to the results of TOPS in Refs. [16, 48].

**Fig. 6:**
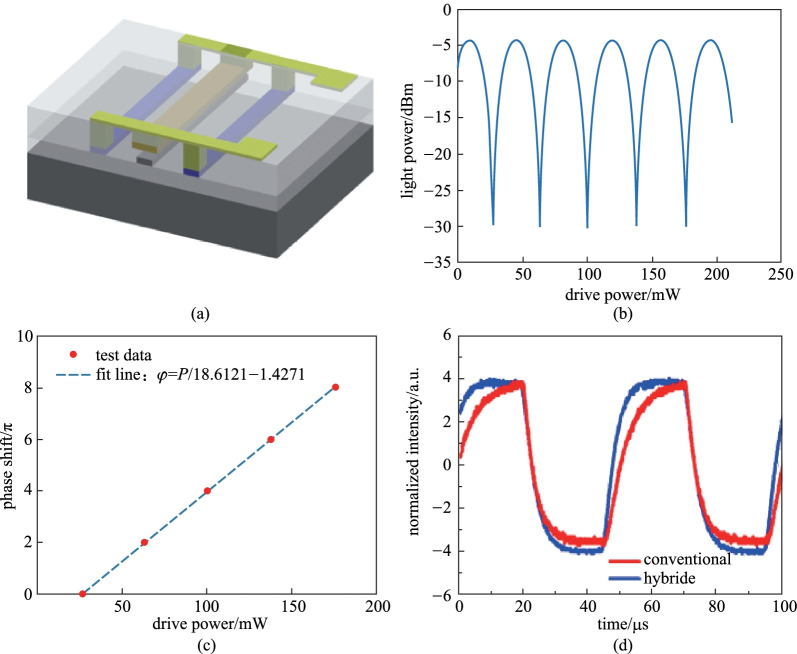
A hybrid TOPS. **a** Schematic of the hybrid TOPS. **b** Output light power versus the drive power. **c** Variation of phase shift versus the drive power. **d** Response time curve of TOPS with the basic structure and hybrid structure

Furthermore, we proposed a hybrid TOPS and fabricated it on the CUMEC silicon foundry platform, as shown in Fig. 6a. Figure 6b is the curve of output power versus the drive power when the TOPS is placed on one arm of the MZI structure. Figure 6c shows the fitting line of the variation of phase shift versus the drive power. The slope of the fitting line denotes the tuning efficiency, which is about 18.61 mW/π. As shown in Fig. 6d, the thermal time constant of TOPS with a hybrid structure is much smaller than the TOPS with a basic structure, without sacrificing tuning efficiency.

### TOPS with the silicon substrate undercut

**Fig. 7:**
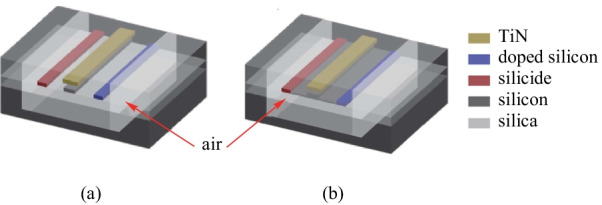
Schematic of TOPS of **a** strip waveguide and **b** rib waveguide with air-gap trench and silicon substrate undercut

The thermal conductivity of air, at 0.31 W/(m·K), is almost three orders of magnitude smaller than that of silicon, 150 W/(m·K). An air-gap trench and silicon substrate undercut post-processing have been chosen to reduce the heat leakage to the environment, as shown in Fig. 7. Table 2 is the experimental results for TOPS devices with silicon substrate undercut which have been designed and fabricated by different organizations. As compared to the TOPS with the basic structure, a significant improvement of tuning efficiency is achieved. However, the thermal time constant is adversely affected due to the reduced heat conductivity by the air-gap trench and silicon substrate undercut. When a TOPS with silicon substrate undercut is applied in one phase arm of the MZI structure the thermal time constant is no less than 266 μs [50, 52–54]. The relationship between thermal time constant and − 3 dB bandwidth (*f*_−3 dB_) [55, 56] can be written as

10 $$\tau { = }\frac{0.35}{{f_{{ - 3\;\,{\text{dB}}}} }}.$$

**Table 2:** Performance list of TOPS with silicon substrate undercut designed and fabricated by different organizations

Heater type	Waveguide type	Test structure	Tuning efficiency/(mW·π^–^^1^)	Heating time/μs	Cooling time/μs	Organization	References
Tungsten	Single strip WG	MZI	1.42	198	227	IMEC	[50]
Silicide	Single strip WG	MZI	1.49	188	218	IMEC	[50]
Doped silicon	Single strip WG	MZI	1.42	151	217	IMEC	[50]
TiN	Single strip WG	MZI	0.49	144	122	IME	[52]
TiN	Single strip WG	MI	0.05	780	500	IME	[53]
Pt	Single strip WG	MZI	0.54	141	NR	OSU	[54]
TiN	Threefolded strip WG	MZI	0.5	640	700	Huawei	[57]
TiN	9-folded strip WG	MZI	0.095	750	1200	UBC	[58]

TiN: titanium nitride, WG: waveguide, NR: not reported, MZI: Mach–Zehnder interferometer, MI: Michelson interferometer

Therefore, the − 3 dB bandwidth of TOPS with air-gap trench and silicon substrate undercut is below 2.6 kHz, which makes them unattractive for several emerging applications.

**Fig. 8:**
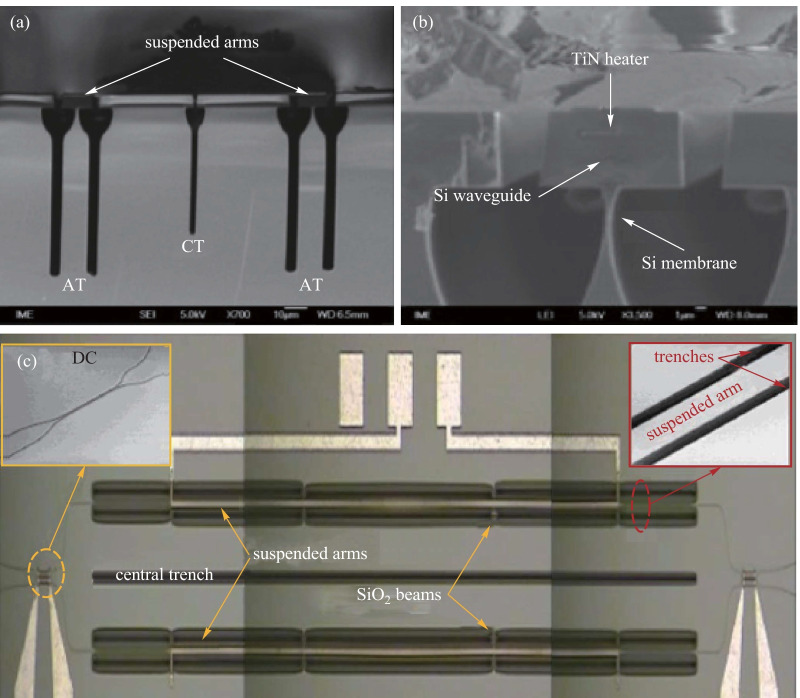
SEM images of TOPS with suspended arms [52]. **a** Cross-sectional SEM images (AT: arm trenches, CT: central trench). **b** Cross-section of the suspended arm. **c** Schematic diagram of 2 × 2 silicon photonics switch with suspended phase arms

**Fig. 9:**
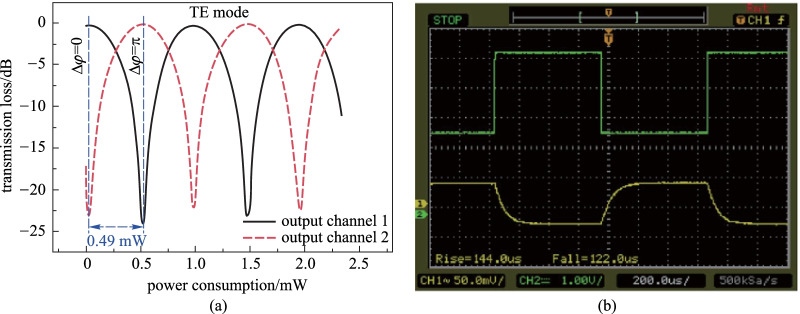
Experimental results. (**a**) Tuning efficiency curve and (**b**) rise time and drop time of TOPS with suspended arms [52]

Except for the structure in Ref. [54], all the other structures shown in Table 2 are fabricated on the SOI platform. After a standard back-end process, an air-gap trench was fabricated by deep-etching down to the silicon substrate. Then, anisotropic selective silicon etching was applied to the silicon substrate to undercut the waveguides [52]. Perspective and cross-section images are shown in Fig. 8. Here, two 4.0 μm wide arm trenches are fabricated on both sides of each TOPS to prevent the heat from leaking into the adjacent SiO_2_ layer. Another central trench is designed at the center of the two arms to further reduce the thermal crosstalk. The transmission loss of the switch for TE mode at 1550 nm as a function of power consumption and the time-domain response characteristics are shown in Fig. 9. The optical crosstalk of this switch is more than 23 dB, and the switching power, i.e., tuning efficiency of TOPS, is only 0.49 mW/π. The 10%–90% thermal time constant is about 266 μs, including the rise time of 144 μs and the fall time of 122 μs.

**Fig. 10:**
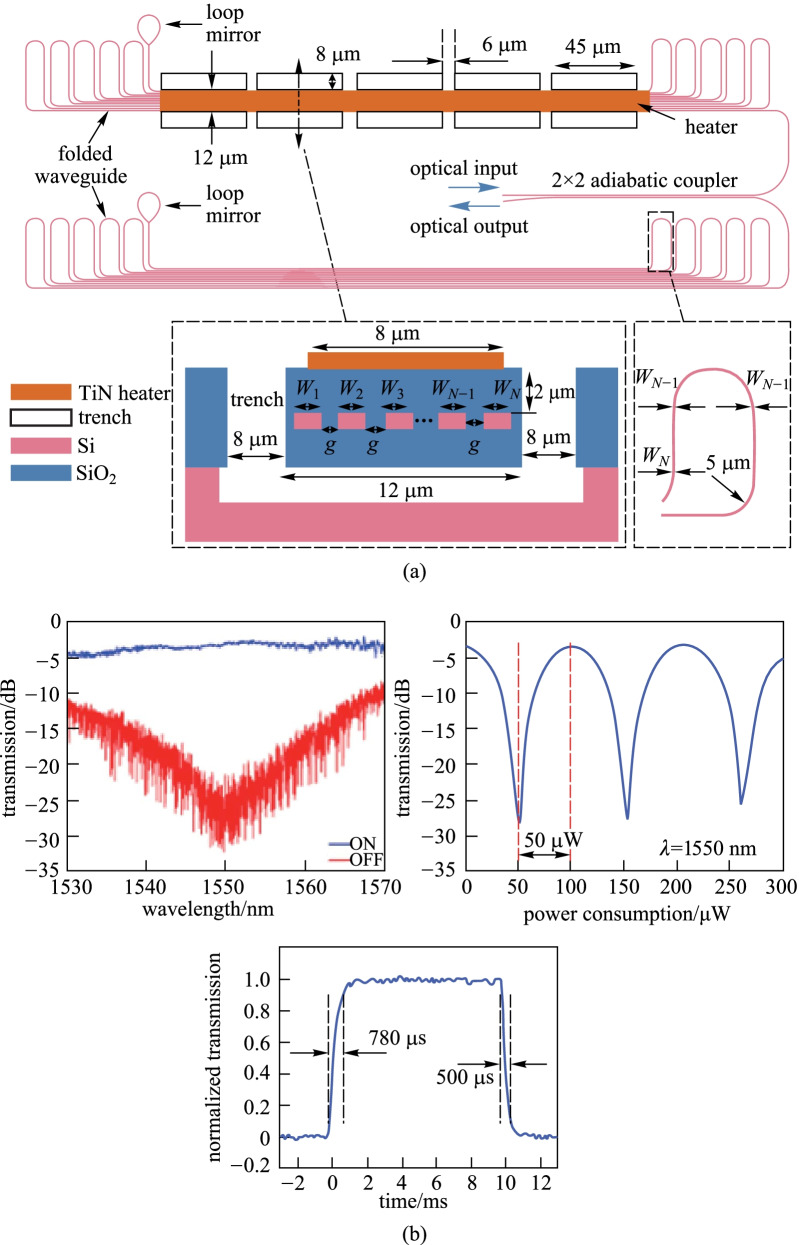
Schematic and experimental results of Michelson interferometer [57]. **a** Schematic of Michelson interferometer based on the TOPS with folded waveguides and suspended structure. **b** Measurement results for the device

To further improve the tuning efficiency of the TOPS, a folded waveguide and suspended structure have been adopted simultaneously [53], as shown in Fig. 10a. The two types of structures are used to increase the optical interaction length of the light with the heated region and improve thermal isolation, respectively. Finally, the tuning efficiency can be improved to 0.05 mW/π, which is an order of magnitude higher than the TOPS with silicon substrate undercut reported in other literature [50, 54, 57, 58]. Here, a Michelson interferometer (MI) has been adopted to replace the MZI [57]. The measurement results have been shown in Fig. 10b, the measured power required to switch from the maximum to minimum transmission is only 50 μW, and the thermal time constant is 1.28 ms, including a rise time of 780 μs and fall time of 500 μs.

**Fig. 11:**
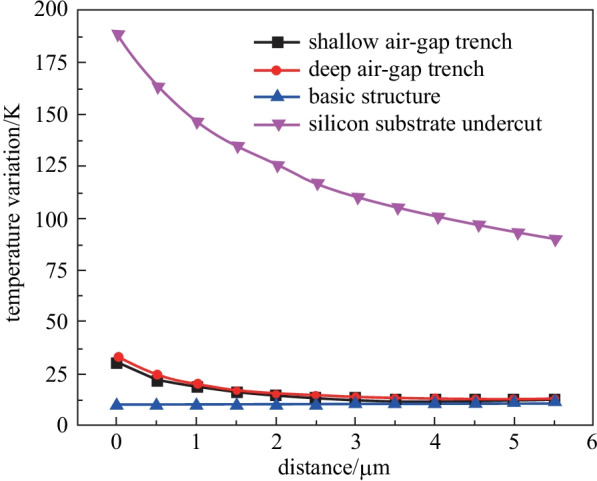
Simulation results of the temperature variation of the waveguide along with the distance between the waveguide and air-gap trench

The process of producing silicon substrate undercut is much more complex than that of producing the basic structure, and this kind of TOPS on our silicon platform is still under development. At the same time, the temperature variation of waveguides versus different structures has been analyzed. As shown in Fig. 11, the temperature variation of the waveguide of TOPS with silicon substrate undercut is much larger than the TOPS with only an air-gap trench and basic structure, which means the tuning efficiency of TOPS with undercut is much higher than the TOPS with only an air-gap trench.

Although the tuning efficiency of TOPS can be improved by creating a vertical air-gap trench and silicon substrate undercut surrounding the silicon waveguide [50, 52–54, 57, 58], some drawbacks have also been caused. First, densely placed air-gap trenches or silicon substrate undercut structures over a large area limits the scalability of integration. Second, the reliability has been reduced due to the accumulated mechanical fatigue from temperature stress. Third, the thermal time constant has increased by about 20 times. Therefore, the TOPS with air-gap trench and silicon substrate undercut is unbeneficial for applications that require fast response, such as optical neural networks, quantum computation devices [15].

### TOPS with a folded waveguide

**Fig. 12:**
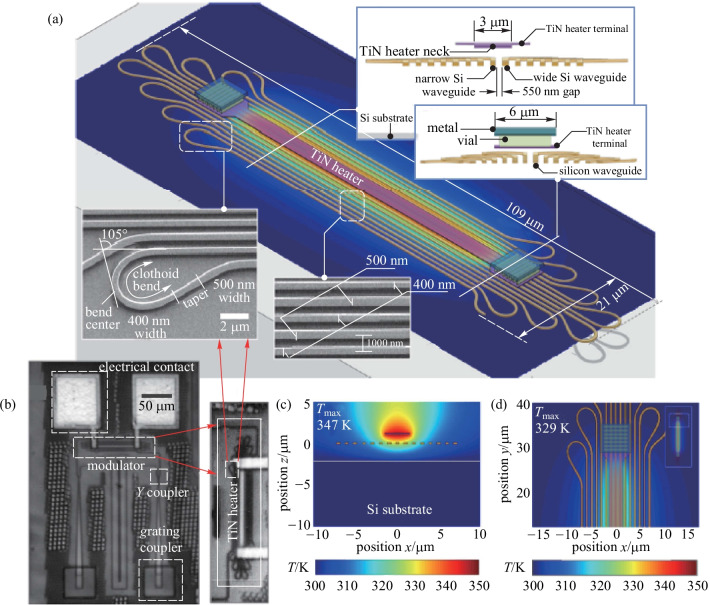
Fabricated structure of a TOPS with geometrical design optimization for tuning efficiency improvement [59]. **a** Physical layout of the fabricated TOPS with SEM photographs of multi-section Clothoid bend structures and waveguide array with alternating widths. **b** Microphotograph of a fabricated TOPS test chip. **c** Vertical and **d** horizontal cross-section of the TOPS with simulated temperature profile at the center of the silicon waveguide

**Fig. 13:**
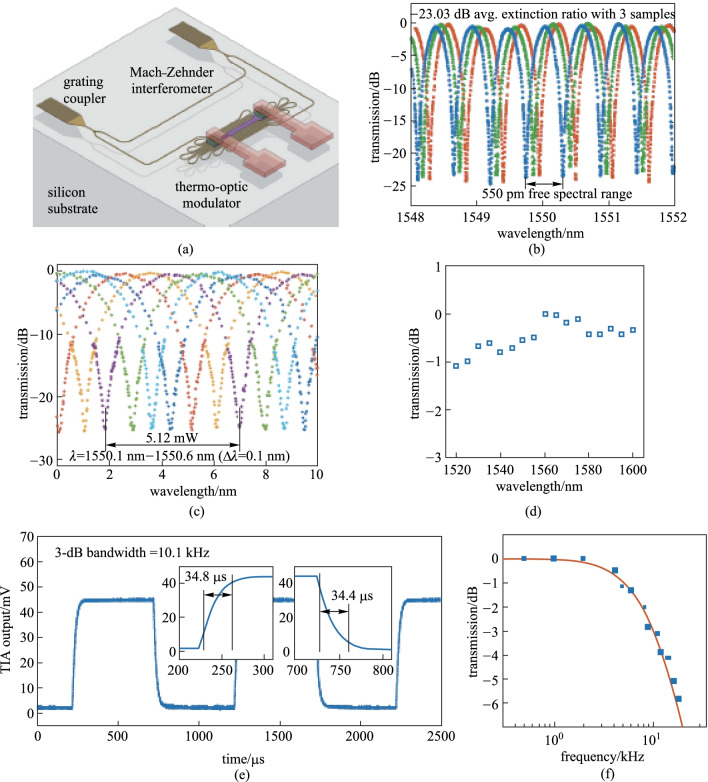
Experimental results of TOPS with the folded waveguide [59]. **a** MZI structure for a test. **b** Spectral of three samples with average extinction ratio –23.03 dB. **c** Tuning efficiency of 2.56 mW/π. **d** Normalized optical transmission. **e** Rise time and fall time of 34.8 and 34.4 μs. **f** Thermo-optic bandwidth test

Due to the fact that the area of heat flow is much larger than the cross-section area of the waveguide, TOPS devices with folded waveguides have been proposed and fabricated [57–59]. Recently, Chung et al. have reported an experimental demonstration of geometrical design optimization for improving the tuning efficiency of a low-loss silicon thermo-optic waveguide phase shifter on a standard silicon photonics platform (see Fig. 12) [59], whose footprint is only 0.0023 mm^2^. The TOPS has been experimentally measured using an on-chip MZI, and the results are shown in Fig. 13. The TOPS consumes 2.56 mW for a π phase shift over 100 nm optical bandwidth while achieving 1.23 dB optical loss. Besides, the − 3 dB bandwidth of the TOPS is about 10.1 kHz. Therefore, the *P*_π_·*τ* product of the TOPS is about 88.57 mW/π·μs.

**Fig. 14:**
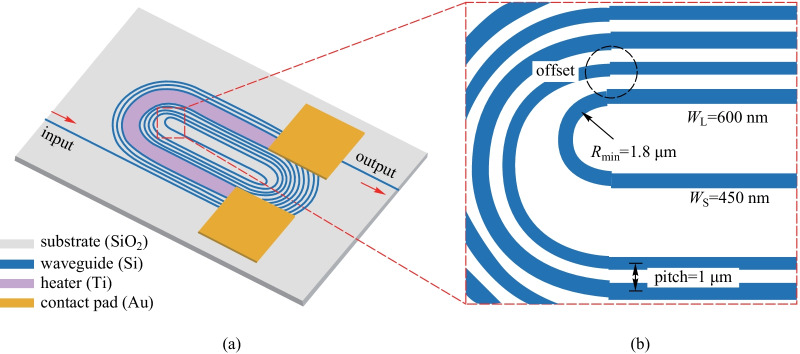
**a** Schematic of the proposed TOPS using a spiral waveguide. **b** Zoom-in the offset part [60]

**Fig. 15:**
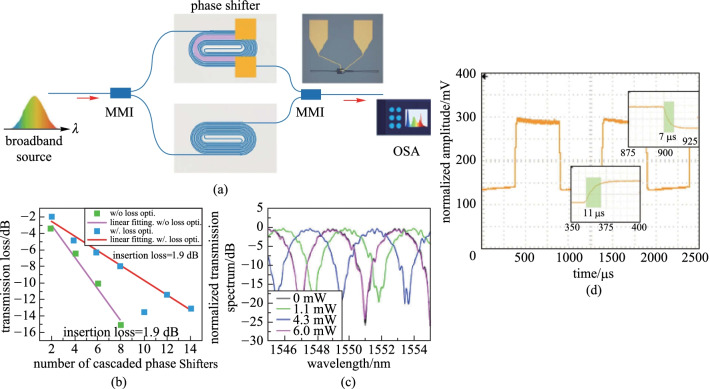
**a** MZI structure for measuring the phase tuning tuning efficiency. **b** Insertion loss of the device with optimization. **c** Spectrum of the MZI with the electrical power varies from 0 to 6.0 mW. **d** Modulation bandwidth of the TOPS [60]

In addition, a TOPS based on a densely distributed silicon spiral waveguide on an SOI platform has been experimentally demonstrated by Qiu et al. [60] (see Fig. 14). The phase shifter shows a well-balanced performance in all aspects. The electric power consumption is as low as 3 mW to achieve a phase shift, the optical insertion loss is 0.9 dB, the footprint is 67 × 28 μm^2^ under a standard silicon photonics fabrication process without silicon air-gap trench or substrate undercut process, and the modulation bandwidth is measured to be 39 kHz, as shown in Fig. 15.

The FOM of the TOPS is about 33 mW/π·μs, which is a benefit for large-scale silicon PICs as an efficient fundamental unit. Compared with the TOPS with silicon substrate undercut, this kind of structure is much easier to fabricate with a lower FOM value. Besides, the tuning efficiency of this kind of TOPS can be adjusted by changing the folded times of the waveguide. Unfortunately, the insertion loss is positively related to the folded times of the waveguide.

**Fig. 16:**
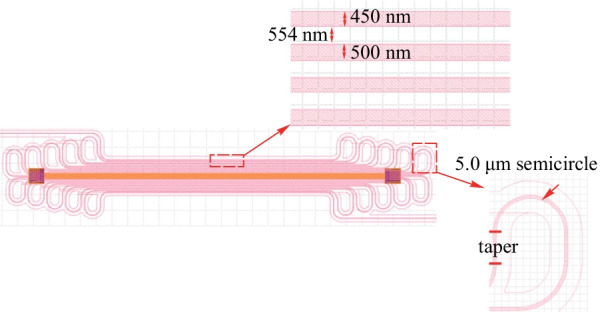
Schematic of TOPS with a folded waveguide fabricated on CUMEC silicon platform

To obtain the optimal folded times of waveguide, we have fabricated TOPS devices with folded waveguide on the CUMEC silicon platform and have analyzed their performances in terms of a new figure of merit (FOM_2_), accounting for the influence of the insertion loss:

11 $${\text{FOM}}_{{2}} = P_{\uppi } \cdot \tau \cdot {\text{IL}},$$

where IL is the insertion loss of the device. As shown in Fig. 16, the widths of the two adjacent waveguides are 450 and 500 nm. The two waveguides are interval distribution with a spacing of 554 nm. There is no mode crosstalk between the two adjacent waveguides because the effective indices of the two waveguides are different. The propagation loss of the bend can be ignored since the radius is much larger than 5.0 μm. The heater is TiN metal and placed on top of the waveguide.

The loss of the device is only determined by the length of the folded waveguide. Therefore, Eq. (11) can be wrn as

12 $${\text{FOM}_{2}} = P_{\uppi } \cdot \tau \cdot {\text{IL}}_{{\text{s}}} \cdot n.$$

**Fig. 17:**
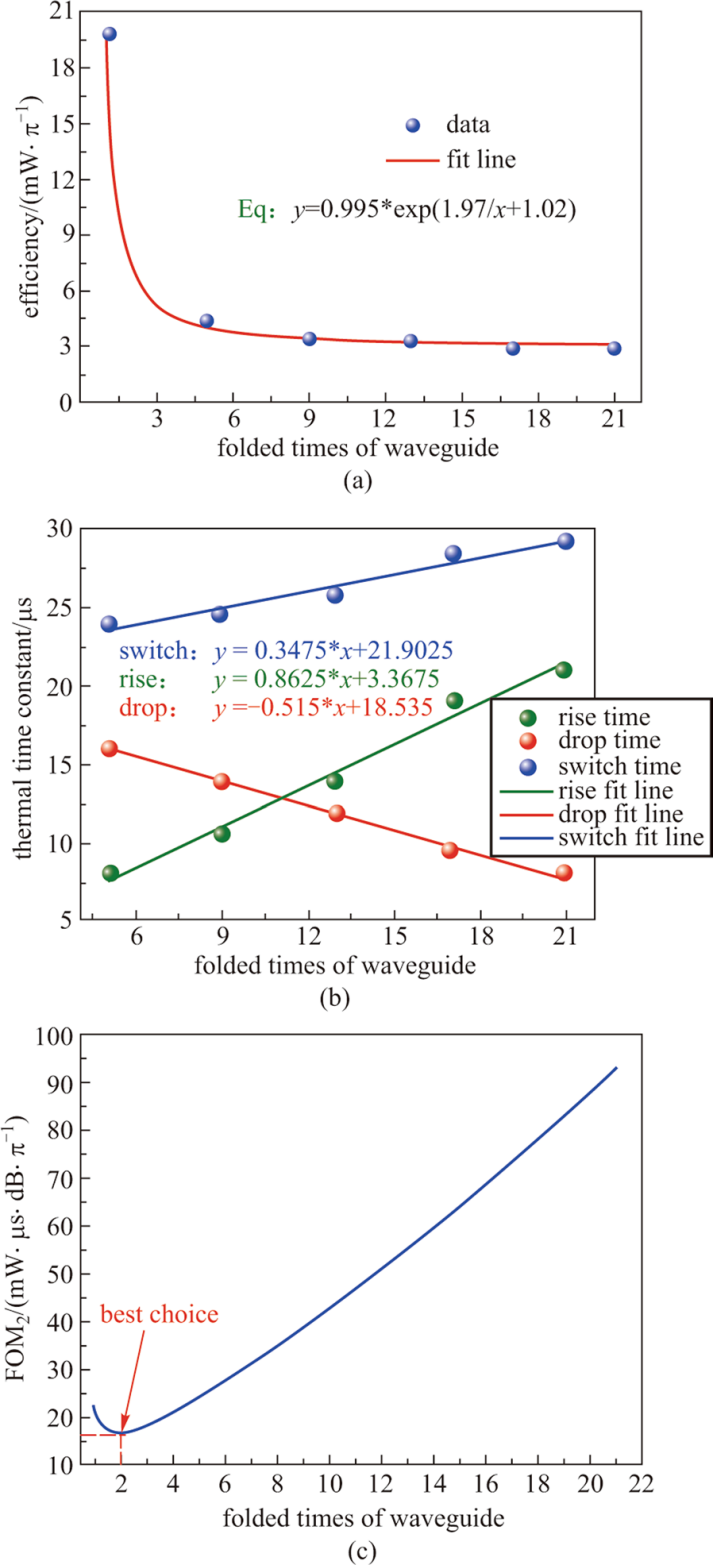
Experimental results of TOPS with folded waveguides, **a** tuning efficiency, **b** thermal time constant, **c** value of FOM_2_ versus with different folded times of waveguide

Here, IL_s_ and *n* are the loss of single folded waveguide and the folded times of waveguide, respectively. As shown in Fig. 17c, when the folded time of the waveguide is 2, the FOM_2_ is smallest. Consideration of the actual layout, a TOPS composed of a threefolded waveguide is a benefit for a large-scale PIC. Figure 17a and b is the curve of the tuning efficiency and thermal time constant versus the number of folds of the waveguide.

### TOPS with a multi-pass waveguide

**Fig. 18:**
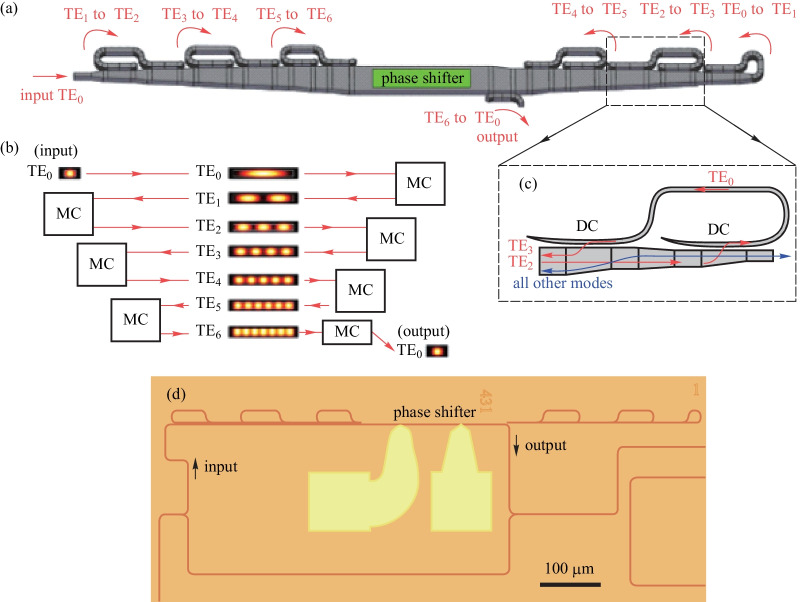
Multi-pass TOPS based on mode multiplexing [61]. **a** Schematic of a seven-pass structure. **b** Schematic description of the light pass. **c** Schematic of a structure that converts the TE_2_ mode to the TE_3_ mode. **d** Optical microscope image of MZI test structure

**Fig. 19:**
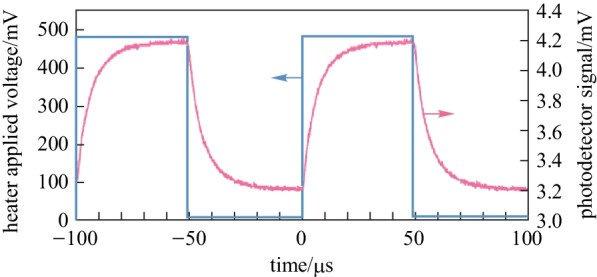
Temporal response of the TOPS with the 7-pass recycling structure. In all 1-pass, 3-pass, 5-pass, and 7-pass devices, the thermal time constant of rise time and fall time are (6.4 ± 0.2) and (6.6 ± 0.4) μs [61]

As is well known, there is always a trade-off between the tuning efficiency and thermal time constant of TOPS. To improve the tuning efficiency of TOPS without sacrificing the thermal time constant, light recycling based on resonators has been employed to improve the utilization tuning efficiency of drive power. As shown in Fig. 18, a multi-pass TOPS that lowers power consumption to 1.7 mW per π phase shift has been experimentally demonstrated [43]. The heater is placed on the top of the waveguide. An tuning efficiency of 15.4, 4.6, 2.6, and 1.7 mW/π are measured in the 1-pass, 3-pass, 5-pass, and 7-pass phase shifter, respectively. This corresponds to a power-tuning efficiency enhancement of 3.3, 5.9, and 8.9 times in the 3-pass, 5-pass, and 7-pass phase shifter, respectively. Note that the factor of enhancement is slightly higher than the number of passes. This is because the effective refractive indices of the higher-order modes are more sensitive to temperature change due to stronger dispersion. A thermal time constant of 6.5 μs is measured, which is independent of the number of passes, as shown in Fig. 19.

**Fig. 20:**
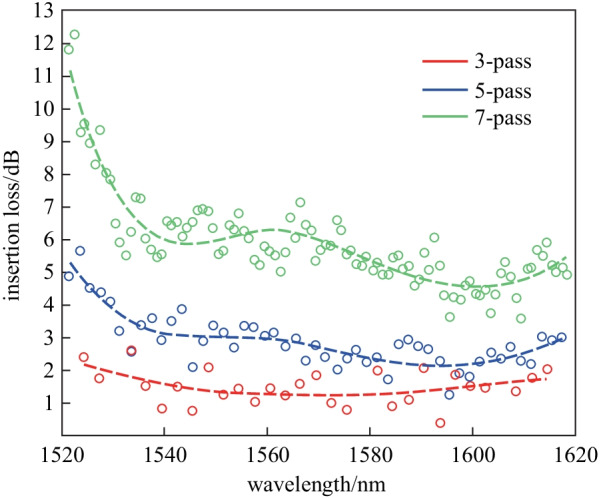
Insertion loss for three-, five-, and seven-pass devices as a function of wavelength, extracted from the MZI transmission spectra [61]

Therefore, the FOM of the 7-pass recycling-enhanced phase shifter is 11.1 mW/π·μs. It is smaller than for other TOPS, including those with the heater on a dense spiral waveguide (FOM = 33 mW/π·μs) and integrated doped-silicon heaters with adiabatic bends (FOM = 30.5 mW/π·μs). However, the insertion losses are about 1.2 dB (at the wavelength of 1570 nm), 2.2 dB (at 1594 nm), and 4.6 dB (at 1601 nm) for the 3-pass, 5-pass, and 7-pass structure, respectively (see Fig. 20).

**Fig. 21:**
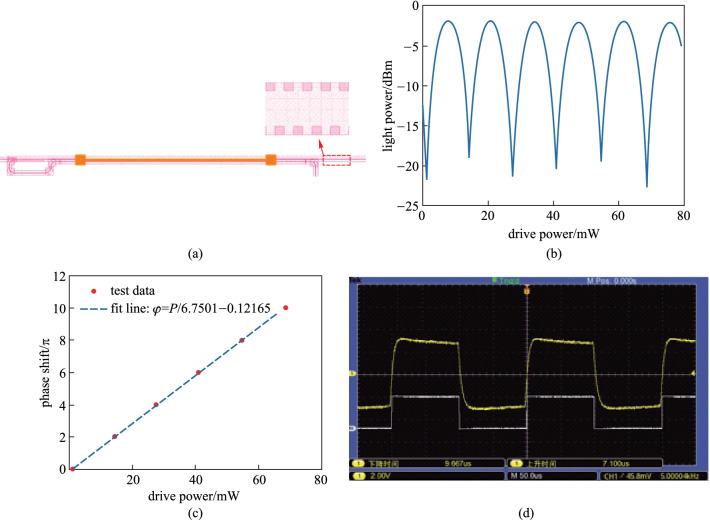
**a** Schematic of TOPS with multi-pass waveguide, the illustration figure is the schematic of the antisymmetric grating. **b** Curve of output light power versus the electrical driving power. **c** Fitting line of phase shift versus the electrical driving power. **d** Response curve of the TOPS is under 5.0 kHz

The disadvantage of this kind of structure is its large footprint and insertion loss. To solve this problem, we propose a new structure, as shown in Fig. 21a. An antisymmetric grating has been adopted to achieve mode conversation between TE_0_ mode and TE_1_ mode, which has a smaller size and lower insertion loss [61]. Unfortunately, the grating used to implement high-order mode conversation is complex. Therefore, the TOPS with only mode conversation of TE_0_ and TE_1_ based on antisymmetric grating has been fabricated on the CUMEC silicon platform. Compared with the TOPS of mode conversation via the asymmetric directional coupler, the footprint of this kind of TOPS is smaller. Moreover, *P*_π_ is closer to one-third of that of the TOPS with one pass waveguide. Significantly, the mode conversion only happens between TE_1_ mode and TE_0_ mode, which can reduce the insertion loss effectively.

### TOPS with the integrated diode

**Fig. 22:**
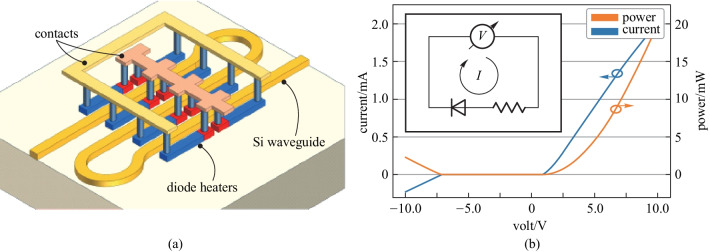
The schematic and experimental results of TOPS with the integrated diode [62]. **a** Phase shifter was implemented with eight diode heaters placed in parallel. **b** *I*–*V* curve showing the diode characteristic of the phase shifter. The inset figure is the equivalent electric circuit of the heater

When the complexity of the large-scale photonic circuit increases, the circuit needs to be driven by means of hundreds or thousands of contact pads and voltage sources. The contact pads always occupy a large space in the photonic chip. Besides, a great number of wires are needed to connect the active devices to the pads. Therefore, the interfacing with electronics for controlling and read-out becomes a limiting factor for the scalability of the system. In recent years, Wim Bogaerts proposed a novel structure of TOPS with an integrated diode [62], as shown in Fig. 22. As mentioned earlier, doped silicon can be used as a resistor material to implement a TOPS on the SOI platform. Instead of either *P-type* or *N-type* dopants to increase the conductivity of the heater, the main body of the heater can use *N-type* dopants, and the region near one of the electrical contacts can be doped by *P-type*, creating a *PN* junction inside the heater. This approach converts the standard heater to a diode in series with a high resistivity strip. The total length of the heater is 50 μm, where 8 μm is used for the *P-type* doped region. The width of the heater is 1.2 μm. The heaters are placed closed to the target waveguide, keeping a gap of 0.75 μm between the heater and the waveguide. The gap was chosen to be close enough to increase the power consumption of the heater yet avoid leaking of the light from the waveguide to the heater. The heater and the waveguide have different widths to minimize coupling due to phase matching. The tuning efficiency of the TOPS is about 15–20 mW/π.

**Fig. 23:**
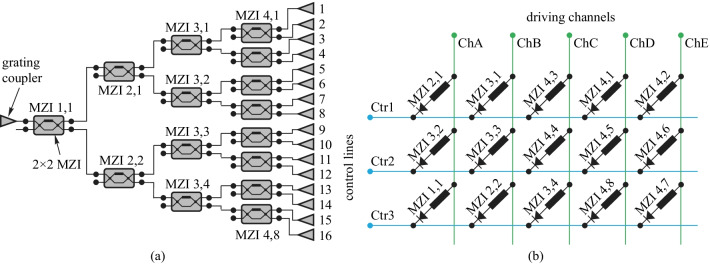
**a** 1 × 16 splitter tree circuit consisting of 15 tunable couplers, each of which is a balanced MZI with a TOPS in one arm. **b** Electrical connectivity of the 15 phase shifters. They contain a diode in series and are connected to three control lines and five driver channels [62]

Furthermore, a diode-based TOPS in a matrix topology, grouping the heaters in sets of *M* columns and *N* rows, has been proposed and experimentally demonstrated, as shown in Fig. 23. In this arrangement, the anodes of the diode-heaters are connected in the same row together, while the cathodes are connected in the same column together. Here, the rows and columns of the matrix are defined as control lines and driving channels. It is possible to address one specific phase shifter in the matrix by setting the voltage level at its correspondent control line at a low level (GND) while setting the voltage of its correspondent driving channel at a high level (*V*+).

**Fig. 24:**
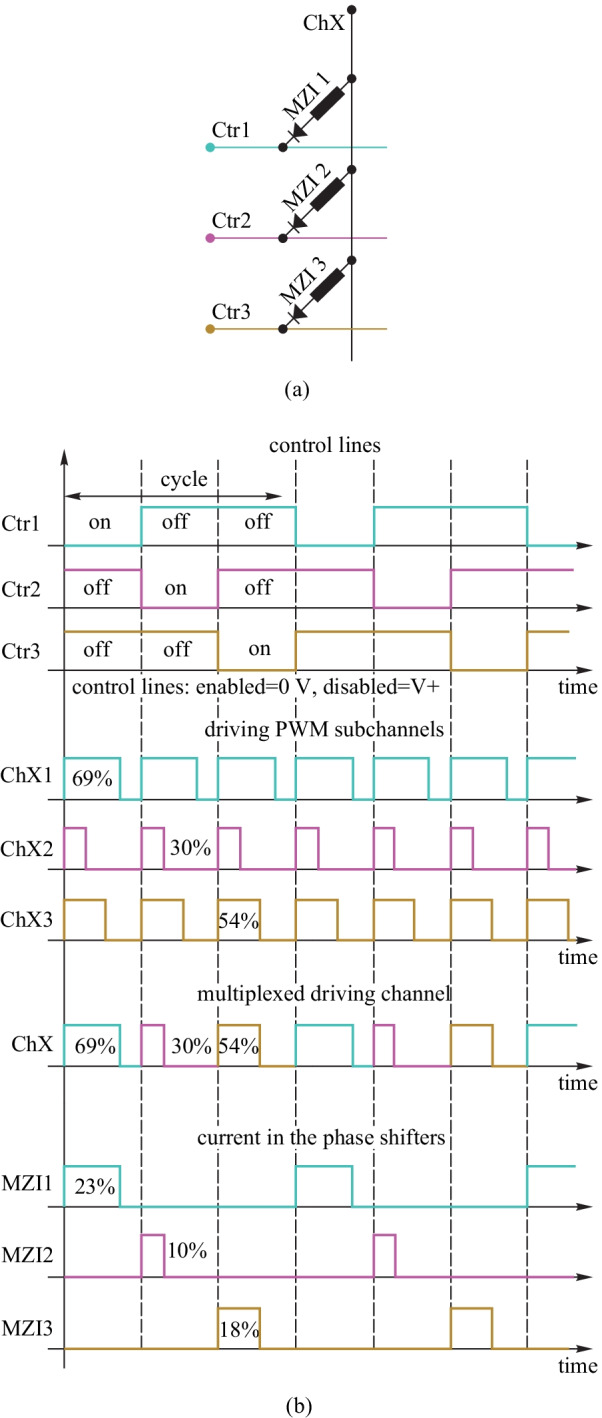
Time multiplexing the signals on the driving channel [62]. **a** A single driving channel ChX connecting three MZIs to control lines. **b** Control mechanism of the sub-circuit

Moreover, the matrix circuit can be divided into five identical sub-circuits along a single column, each containing one driving channel and three control lines, as shown in Fig. 24a. This requires a total of eight contact pads (and pulse width modulation (PWM) driving sources) to drive all 15 phase shifters needed to operate the circuit simultaneously. Figure 24b shows the time traces of a single driving channel in a circuit with *N* = 3 control lines.

In addition, time multiplexing has been adopted to improve the flexibility of this method. 1/*N*, where *N* is number of control lines, of the total cycle can be used by a TOPS at the same time. Therefore, the number of bond pads and power sources of a matrix arrangement that enables the driving of *N* × *M* phase shifters are (*N* + *M*) by using the PWM signal to implement multiplexed control. This technique is especially useful in silicon PICs with many TOPS devices but without enough space for electrical connections.

## Discussion

As described above, there are many kinds of TOPS devices that use the SOI platform. Each kind of TOPS has advantages and disadvantages. To allow researchers to better choose the type of TOPS according to their demands, the performances of some typical TOPS are listed in Table 3. It is worth noting that the TOPS designed by the fabless organizations is not represented in Table 3.

**Table 3:** Performance list of typical TOPS

TOPS type	Silicon substrate undercut	Tuning efficiency/(mW·π^–^^1^)	Heating time/μs	Cooling time/μs	FOM/(mW·π^–^^1^·μs)	Footprint/(μm × μm)	Foundry	References
Doped silicon	N	21.96	12.80	2.60	169.09	3.65 × 200	CUMEC	[49]
Silicide	N	21.75	12.90	4.00	183.79	3.65 × 200	CUMEC	[45]
TiN	N	19.12	5.90	8.97	142.15	2.0 × 200	CUMEC	[45]
Folded waveguide	N	2.75	21.1	8.1	40.15	59.0 × 278	CUMEC	[45]
Multi-pass waveguide	N	6.75	6.2	6.6	43.2	18.3 × 450	CUMEC	[45]
Hybrid	N	18.61	6.0	6.0	111.66	3.65 × 200	CUMEC	[45]
Doped silicon	N	20.4	21.3	66.0	890.46	–	IMEC	[50]
Silicide	N	22.5	19.1	75.8	1067.6	–	IMEC	[46]
W	N	23.4	38.2	45.1	974.61	–	IMEC	[46]
Doped silicon	Y	1.30	236	156	254.8	–	IMEC	[46]
Silicide	Y	1.49	188	218	302.47	–	IMEC	[46]
W	Y	1.42	198	227	301.75	–	IMEC	[46]
TiN	N	21.4	5.6	NR	119.84	7.5 × 320	AMF	[15]
Doped silicon	N	22.8	2.2	NR	50.16	4.1 × 320	AMF	[15]
Silicide	Y	20	2.8	2.2	50	3.5 × 200	IBM	[16]
Doped silicon	Y	24.77 ± 0.43	NR	NR	NR	3.0 × 61.6	OpSIS	[51]
TiN	Y	0.49	144.0	122.0	66.5	14 × 1000	IME	[52]

TiN: titanium nitride, W: tungsten, N: no, Y: yes, NR: not reported

As shown in Table 3, a heater in the TOPS is generally made of metal, doped silicon, or silicide on the SOI platform. For a TOPS with a basic structure, the tuning efficiency is about 20 mW/π. When the PIC consists of more than 1000 TOPS of this configuration, the power consumption would be more than 20 W. To reduce power consumption, further improvements should be made to improve the tuning efficiency of TOPS devices. Silicon substrate undercut is an effective way to improve the tuning efficiency of TOPS devices since the heat generated by the heater is mainly accumulated in the vicinity of the waveguide and does not leak to the environment. The tuning efficiency can be improved to about 1.50 mW/π, which is less than a tenth of that of a TOPS without silicon undercut. However, the thermal time constant of this structure is about 200 μs, which is unbeneficial for large-scale PICs. The requirement for TOPS devices for large-scale PICs, such as optical neural networks and optical phased arrays, are high tuning efficiency and fast switching time, i.e., small FOM. Moreover, a TOPS with a folded waveguide and multi-pass waveguide has been experimentally investigated to meet these needs. Unfortunately, the two kinds of TOPS would cause a higher insertion loss, which greatly limits the scale of an optical neural network or optical phased array. It is worth mentioning that the TOPS of a hybrid structure can improve the thermal time constant without sacrificing tuning efficiency and increasing insertion loss. This kind of TOPS has not been widely used in PIC since the improvement is not obvious. Besides, the thermal crosstalk effect can strongly affect the application of TOPS on PIC. To solve this problem, many approaches have been adopted, such as optimizing the chip layout, isolating thermal diffusion, developing temperature-insensitive devices, and packaging with a thermo-electric cooler (TEC).

Note that, high phase tuning efficiency is the requirement for all applications for TOPS devices. When the TOPS is used for adjusting the working point of the photonic device, such as the Mach–Zehnder modulator, fast switching time is not necessary. However, when the TOPS is used in reconfigurable silicon photonic circuits, such as optical neural networks, optical-path-routing switches, optical phased arrays, quantum processors, and programmable photonic circuits, high tuning efficiency and fast switching time are required at the same time. Furthermore, the scale of these circuits is closely related to the loss and footprint of TOPS. In summary, the TOPS with high phase tuning efficiency, fast switching time, low loss, and small footprint, is very promising for various applications on the SOI platform.

## Conclusion

This work provides an overview of various TOPS devices on the SOI platform, together with a brief theoretical explanation and a review of the TOPS devices fabricated on different silicon foundry platforms. Compared with other foundries, the CUMEC silicon platform can provide both design and fabrication of all these TOPS devices at the same time. Low loss, small thermal time constant, higher phase tuning efficiency, and addressable TOPS devices are requirements for achieving further development.
